# A qualitative study of changes in expectations over time among patients with chronic low back pain seeking four CAM therapies

**DOI:** 10.1186/s12906-015-0531-9

**Published:** 2015-02-05

**Authors:** Emery R Eaves, Karen J Sherman, Cheryl Ritenbaugh, Clarissa Hsu, Mark Nichter, Judith A Turner, Daniel C Cherkin

**Affiliations:** Group Health Research Institute, Seattle, USA; Department of Family and Community Medicine & School of Anthropology, University of Arizona, Tucson, AZ USA; Department of Psychiatry & Behavioral Sciences and Department of Rehabilitation Medicine, University of Washington School of Medicine, Seattle, WA USA

**Keywords:** Chronic low back pain, Acupuncture, Chiropractic, Yoga, Massage, CAM, Expectations, Self-care, Pain management

## Abstract

**Background:**

The relationship between patient expectations about a treatment and the treatment outcomes, particularly for Complementary and Alternative Medicine (CAM) therapies, is not well understood. Using qualitative data from a larger study to develop a valid expectancy questionnaire for use with participants starting new CAM therapies, we examined how participants’ expectations of treatment changed over the course of a therapy.

**Methods:**

We conducted semi-structured qualitative interviews with 64 participants initiating one of four CAM therapies (yoga, chiropractic, acupuncture, massage) for chronic low back pain. Participants just starting treatment were interviewed up to three times over a period of 3 months. Interviews were transcribed verbatim and analyzed using a qualitative mixed methods approach incorporating immersion/crystallization and matrix analysis for a decontexualization and recontextualization approach to understand changes in thematic emphasis over time.

**Results:**

Pre-treatment expectations consisted of conjecture about whether or not the CAM therapy could relieve pain and improve participation in meaningful activities. Expectations tended to shift over the course of treatment to be more inclusive of broader lifestyle factors, the need for long-term pain management strategies and attention to long-term quality of life and wellness. Although a shift toward greater acceptance of chronic pain and the need for strategies to keep pain from flaring was observed across participants regardless of therapy, participants varied in their assessments of whether increased awareness of the need for ongoing self-care and maintenance strategies was considered a “positive outcome”. Regardless of how participants evaluated the outcome of treatment, participants from all four therapies reported increased awareness, acceptance of the chronic nature of pain, and attention to the need to take responsibility for their own health.

**Conclusions:**

The shift in treatment expectations to greater acceptance of pain and the need for continued self-care suggests that future research should explore how CAM practitioners can capitalize on these shifts to encourage feelings of empowerment rather than disappointment surrounding realizations of the need for continued engagement with self-care.

## Background

Use of complementary and alternative medicine (CAM) is common among chronic low back pain patients [1]. In addition to increasing clinical research that shows pain reduction resulting from CAM [2-5], CAM therapies are often associated with additional benefits [6,7]. Although nearly forty percent of adults in the U.S. use some form of CAM [8], the motivations for people to choose CAM are not well understood. Bishop et al. [9] found that continued use of CAM was related to positive experiences with CAM, rather than with negative experiences or perceptions of conventional care. Despite inconsistent findings, many researchers have continued to argue that higher patient expectations of treatment benefits from CAM therapies enhance treatment effects for a variety of health conditions [10-14]. Further research is needed to understand the relationship between patients’ expectations for treatment, practitioners’ presentation of treatment, and outcomes or reports of satisfaction by patients [15-18]. Further, recent research differentiating hope from expectations has called for a re-examination of how patients themselves perceive their expectations of therapy and how these perceptions change over time in the context of a novel treatment experience [19-22].

Initial research has shown a correlation between use of CAM and high internal health locus of control (HLOC) and empowerment [23]. Murthy et al. [1] raise the possibility that CAM users are more engaged in their health care than others because they are already aware of the importance of maintaining their own health. However, questions remain regarding whether individuals entering CAM are more empowered and engaged in their own health prior to seeking treatment, or whether CAM therapies are involved in empowering patients to take a more active role in their health [1]. Evans et al. [24] report that “self-care talk” is interwoven through CAM consultations. Self-care recommendations were co-constructed by clients and practitioners, in particular because the therapeutic relationship includes patients as equal partners in their own care [24,25].

Emerging evidence suggests that CAM has the potential to enhance patients’ awareness of the interconnections among mind, body, and lifestyle and therefore to encourage patients to assume personal responsibility for their health and adopt more effective coping strategies [7,26,27]. In a review of responses to open-ended questions collected in several CAM clinical trials, Hsu et al. [6] found that participants reported outcomes such as increases in hope, relaxation, feelings of empowerment, body awareness, and ability to cope with back pain. Likewise, participants in cancer treatment trials reported that CAM use led to improvements in quality of life, ability to cope with stress, and increased sense of personal control over health [28]. In a study of individuals seeking CAM, Bann et al. [29] found that patients’ reports of increased empowerment and reductions in symptoms were attributable to the patient-centered care and provider support that are particular strengths of CAM therapies. Williams-Piehota et al. [30] call for further research to determine whether CAM, and in particular, interaction with CAM practitioners, may contribute to behavior change.

Sherman et al. [15] reported that patients’ predictions about the outcomes of acupuncture treatment became stronger predictors of treatment outcome over time, suggesting that participants revisit their expectations based on actual experience. Researchers exploring expectations among patients with chronic low back pain have reported that patients often prefer to disclose hopes, while being more cautious about disclosing expectations [20,22,31]. Bishop et al. [9] found participants often unwilling to commit to an evaluation of treatment, preferring to remain open. Being open to the future, retaining hope, and allowing for the possibility of improvement regardless of prognosis is common in chronic illness narratives [19,32]. We have elsewhere reported that hopes are multifaceted and influence the way participants evaluate and report on their expectations and experiences of treatment [21]. Therefore, to explore changes in expectations over time without consideration of hope in the lives of participants would lead to a partial understanding of the way experiences are evaluated and expectations are updated over time.

In describing illness experiences, participants are continually revising expectations about the future as well as reconstructing the past and present to account for illness [33-35]. Hope is experienced in multiple forms, from cognitive and rational to deeply embodied states of being, sometimes transcending everyday reality [21,36]. While expectations are constrained by a reality and rationality that may be felt as overly constrictive in the lives of chronic pain sufferers, “hoping appears to approach reality as something-in-formation, something-in-process, with the implication that one’s own experiences to date are not the last word about oneself and reality, but that experience itself is still in process, with the possibility of a discovery to be made or a novelty to occur” [37].

In this qualitative analysis, we explore individuals’ expectations and hopes as they change over time in the context of CAM treatment for chronic low back pain.

## Methods

Qualitative interviews were conducted as part of a larger study of individuals with chronic low back pain seeking one of four CAM therapies (yoga, massage, chiropractic, acupuncture). See [22,38] for additional information on overall study design and methods and Hsu et al. [22] for detailed participant demographic and therapy use characteristics. In analyzing interviews with CAM practitioners in an earlier phase of this study, Schafer et al. [38] reported that CAM practitioners (specifically acupuncturists, yoga teacher, chiropractors, and massage therapists) tried to help their clients have more realistic expectations of their treatment. Further, because participant expectations could change over time, the practitioners had continually managed them to enhance engagement and satisfaction with treatment. In an analysis focused on the pre-treatment interviews included here, Hsu et al. [22] reported that participants were reluctant to express optimistic expectations and often spoke interchangeably about their hopes and expectations. Expectations were modest, and centered around four main themes: pain relief, improved function, and less frequently physical fitness (primarily among yoga participants), and overall wellness. In this analysis, we include both baseline and follow-up interviews to examine how expectations change over the course of CAM treatment. The analysis presented here focuses on semi-structured open-ended qualitative interviews with 64 participants (23 in Tucson, AZ and 41 in Seattle, WA). Interviews were conducted with two categories of participants. One group had not yet begun or had only recently begun a new treatment. They were enrolled as “longitudinal participants” and were interviewed at additional timepoints after beginning treatment. Each interviewer conducted all three interviews with a given participant. A second group of participants who had already completed or were long-time users of one of the therapies were interviewed one time only and enrolled as “cross-sectional” participants. For interview timing and numbers of participants in each group, see Table 1. Eighteen participants who were enrolled longitudinally did not complete follow-up interviews. We reviewed their pre-treatment comments to make sure they were comparable to those with follow-up data, however, those participants who have no longitudinal component are not included in Table 1.

**Table 1:** **Interview timing based on experience with CAM therapy**

**Participants**	**n**	**Experience with therapy**	**# of interviews**	**Timing of interviews**
Longitudinal	23	Not yet begun CAM therapy	3	Prior to treatment
				After 1–2 weeks of treatment
				3 months after beginning treatment
	8	Recently begun CAM therapy	2	Shortly after starting treatment
				3 months after beginning treatment
Cross-sectional	15	Previously tried or longtime user of CAM therapy	1	Post-treatment or after long-term use for continuous users

We recruited adults with chronic low back pain between the ages of 20 and 70 years, with at least 3 months of pain, and who rated their average back pain in the past week as 3 or higher on a 0 (pain is not at all bothersome) to 10 (pain is extremely bothersome) scale. Recruitment methods included: posters and brochures placed in clinics and yoga studios; online classified advertisements; and CAM practitioner referrals of new patients. In an effort to recruit a diverse sample, we also recruited individuals seeking care from a community clinic that offers massage and acupuncture treatments at no cost to patients and serves a primarily low-income, non-English speaking clientele. With the exception of those interviews, which were conducted in a private room in the clinic, all interviews were conducted by telephone. Four experienced interviewers, two of whom were fluent in Spanish, conducted interviews for the study. Five interviews were conducted and transcribed in Spanish and then translated into English. No differences other than slight variations in definition of terms were evident in Spanish language interviews. Exploration of the different meanings of expectation and hope in Spanish versus English is beyond the scope of this analysis.

Interview guides for longitudinal interviews were designed to elicit a broad range of information related to expectations, hopes, and beliefs about illness. Interview 1 (pre/early treatment) contained questions focused on: treatment history; pain severity and variability; expectations and reasons for trying therapy; expectations for broader life change; provider choice; difference between hopes and expectations; overall attitude and approach to life; stress and other life issues; and impact of pain on life. Interview 2 (after 1–2 weeks) contained questions focused on: changes in pain; initial impressions and experiences of treatment; experiences of surprise; changes in expectations; changes in hopes; stress and other life issues; self-care recommendations given by practitioner and intent to follow-through; and changes in thinking about pain or about life overall. Interview 3 (after 3 months) contained questions similar to interview 2, with increased focus on experience of relief; overall impressions of treatment; experiences of surprise; current expectations (for pain relief, better function, continuing treatment); changes in hopes; and intent to continue with self-care recommendations after treatment. Self-care recommendations from practitioners were explicitly included in the 2^nd^ and 3^rd^ interviews, but not clearly probed in the first interview. The cross-sectional interview (conducted once, shortly after first treatment) guide was derived from all the longitudinal interview guides to cover all relevant topics. Interviews varied in length from 20 to 45 minutes and were audio-recorded and transcribed verbatim. Study protocol and interview guides received approval from the Institutional Review Boards at Group Health Research Institute in Seattle and The University of Arizona in Tucson. All participants provided informed consent.

### Analysis

Interview transcripts were coded using qualitative data analysis software ATLASti [39] (for detailed description of codebook development see Hsu et al. [22]). The present analysis is focused on participants’ reported expectations regarding pain, function, impact on life overall, and hopes, as well as indications of surprise, disappointment or satisfaction with CAM therapies. Qualitative data are useful for providing rich descriptions of complex phenomena [40] that can provide depth and understanding to issues not amenable to quantitative methods [41]. In reporting qualitative data, illustrative quotes are used to support observations and illustrate emergent themes [42].

We used a mixed-methods approach to qualitative data analysis. All transcripts were initially read by the research team and coded for a few key themes to gain a sense of the data. Later, more in-depth codes were determined through an immersion/crystallization approach [43]. After initial coding, we used an open-ended process of matrix analysis [44] to analyze changes in expectations within subthemes as well as changes in thematic emphasis over time. Matrix analysis was conducted as a process of decontextualization and recontextualization. This approach allows quotes of interest to be lifted out and examined more closely to observe change over time. Quotes are then recontextualized into the broader data set to make sure observations agree with the overall context and content of transcripts [43].

In the matrix analysis process, we compiled participant quotes into a grid or “matrix” with rows reflecting themes of interest and columns reflecting interview date. For each participant, the matrix included five themes of interest and two or three interview columns (dependent on number of interviews). For cross-sectional participants, the two columns distinguished between whether participants were referring to initial or current expectations. “Matrices” allowed us to observe change in each theme over time in participants’ narratives, as well as changes in thematic emphasis over time (as illustrated in Tables below). To avoid potential limitations of this approach, cells could contain multiple quotes and quotes could be located in more than one cell. Data were then recontextualized by referring back to code reports and individual transcripts to corroborate observations.

Although the intention of our analysis was to focus on changes in expectations over time, we found that hopes were not separable from expectations in our participants’ narratives. Due to engagement of the two concepts, we pay attention to the role of each in the results presented below. Likewise, we consider self-care, empowerment, and lifestyle impacts, as these emerged as central themes in post-treatment interviews. Quotes are identified with pseudonyms and which interview they pertain to.

## Results

### Study participants

Of the 64 participants, seventy-five percent were women. Sixty-five percent were Caucasian and 12 percent Hispanic. 15 participants were seeking chiropractic, 18 were seeking acupuncture, 13 were seeking massage, and 18 were seeking yoga. See Hsu et al. [22] for detailed participant characteristics.

### Overall observations

Over the course of three interviews we noted differences in participants’ emphasis. In pre- or early-treatment interviews, we flagged any mention of hoping for relief, cure, or reduction in pain level. Some said they had “no expectations” or were “not expecting much”. Others reported they were not sure what to expect. In final interviews, expectations and emphasis were more oriented toward lifestyle and whole person connections. Participants were increasingly aware of the relationship between their bodily well-being and their social well-being, physical activity, diet, and overall lifestyle factors. They reported greater awareness of the chronic nature of their low back pain and greater acceptance of the ongoing nature of a need for pain management and self-engagement. Regardless of reduction in pain, almost all participants mentioned they now understood that continued maintenance activities would be required.

Quotes below are presented by therapy to illustrate patterns of change over time for each and to show overall similarities. In qualitative analysis of participants’ descriptions of the lived experience of chronic pain, there is often a tension between identifying patterns and similarities among participants while providing a fair representation of the complexities and particularities of individual experience [34]. While the four participants in Table 2 are presented for their illustration of the overall trend we observed, we emphasize that individual narratives are complex, non-linear, and at times lacked internal consistency. These issues will be explored in later sections.

**Table 2:** **Changes over time in expectations and hopes for improvement: quotations for each type of treatment**

**Time and therapy**	**Quotation**	***Summary***
***Nora (Chiropractic participant, Seattle)***		
Pre-treatment	I’m hoping that I’ll feel better. I’m hoping that long term that this will lessen my pain and give me a better quality of life. That’s what I’m hoping for. But I’m not going in with an expectation that this is what’s going to happen. DC-124-1	*Modest hopes for less pain and better quality of life; No expectations expressed*
2 weeks later	I’m not even going to go there [to talk cure] until I have a couple more adjustments. It may and that would be so wonderful. And if it doesn’t, if I -- I can live really happily where I am right at this point. DC-124-2	*Substantial improvement in pain but still hesitant to hope for too much; Says she is satisfied with current level of pain*
3 months later	So my expectations are that as long as I kind of tow the line and do the things that I need to do and be responsible … My expectation is that I will be able to stay out of pain as long as I’ve got my brain and I can move around. DC-124-3	*Expectations for sustained improvement with continued maintenance and self-care*
***John (Acupuncture participant, Seattle)***		
Pre-Treatment	Bottom line is that I want to be relieved of the pain that I have. I would say I don’t have expectations beyond the current pain. In other words I’m not going into this thinking that as a result of the treatment I’m no longer going to have back pain. LAC-123-1	*Expectations for relief of current pain; Not expecting to get overall complete cure forever*
2 weeks later	I was not expecting [diet and other recommendations]. And so it had more to do with lifestyle change and the way that my eating habits… I think [acupuncture has] changed my outlook on how I manage my health. Which ultimately affects my back. LAC-123-2	*Surprised by recommendations for self-care, diet changes; Changed outlook on health management*
3 months later	I just know that this is a weakness in my lower back that I’m probably going to have with me and I just got to be much more aware of it and deal with it and not wait ‘til I can’t walk… [the practitioner] helped me be much more aware of what I need to do to keep this under control and to manage my life and the stress in my life. LAC-123-3	*Focus on management of stress and awareness of underlying issues and need for continued maintenance*
***Sarah (Massage participant, Seattle)***		
Pre (early)-treatment	Oh, I thought it would definitely get better. I was really assuming that I would have, you know, less pain and that maybe it would take a number of treatments, but that eventually it would help alleviate the problem… I was hoping [it would cure]. LMP-137-1	*Expectation of less pain; hoping for a cure for pain*
2 weeks later	Well, I think it will get better. I do have expectations that it will get better, but I also think I have to be a lot more cognizant of the way that I use my body. And not be as, you know, careless. LMP-137-2	*Expectation of improvement in pain; increased awareness of need to be more attentive to body*
3 months later	I think it might be a lifelong treatment, although probably not as frequently once I get on top of it. You know? But I think it’s definitely going to be, maintenance is gonna be just part of the whole scene. LMP-137-3	*Shift to seeing maintenance of activities to address back pain as lifelong*
***Jeanne (Yoga participant, Seattle)***		
Pre-treatment	I think it will give me tools to kind of control it, more tools to enable my body to be aware of some of the different muscles or areas or maybe things I shouldn’t do to it, to help control the pain or also learn different things that maybe can relieve it [so]… it’s not causing the pain. YI-132-1	*Expectation of tools to help control or relieve pain (or cause of pain)*
2 weeks later	I feel that there has to be a lot more that we’re just not doing yet, but I don’t know, I just have to get into that routine and they make it simple enough you can do it at home using things you have at home, so I’m kind of expecting even if I were to only take class for a few months, it would have given me some tools and knowledge to keep doing it at home. YI-132-2	*Expecting tools and knowledge to continue at home*
3 months later	I took it for a while and then I stopped taking it for a while, I had too many things that came up and I … I noticed kind of a big, big backslide difference again. So, … so I’ve kind of found that I kind of have to make it part of my routine if I’m gonna feel comfortable… At least I have some tools now, I can use to help. But I have to be the one using the tools. YI-132-3	*Realization that yoga has to be part of routine maintenance; Having tools is only the first step, need to use the tools to experience benefit*

### Becoming more “realistic”

Becoming more “realistic” was a common theme in participants’ narratives. Although a few participants did report significant pain relief as a result of treatment, a more common outcome of treatment was an increased sense of being able to take control of one’s pain, to manage it and to tend to one’s own bodily well-being in a broader sense. Nora, below, offers a metaphorical representation of learning to care for her body as one would care for a garden.

*I really thought that this was what I was tied to. You know, I was tied to this pain. That this pain was definitely a part of me and was going to be a part of me forever. And I don’t think that the chiropractic work heals my back in the sense that, you know, we do some adjustments for five or six months and you’re better and you’re never going to have any discomfort in your back again. I think what it has done for me is that it has showed me that there are really good avenues to take care of your body. You know, besides anti-inflammatories and things like that. That it is -- it’s caretaking, it’s tending, you know, like you would tend to a garden. You know, you can plant all those wonderful tomato plants and lettuce but if you don’t weed it and water it and fertilize it, they’re not going to grow and flourish. So the chiropractic work is part of me tending my body. And if I don’t do this, my body isn’t going to flourish and grow and feel good. And bloom. (Nora, Chiropractic participant, Seattle, Interview 3)*

Here, emphasis has shifted from intense focus on pain to increased sense of bodily awareness and attention to more comprehensive care for the self both in relation to and independently of pain. This sense of shift toward a broader focus on the body, long-term well-being, and empowerment to take control over one’s own search for wellness was central to participant narratives both three months into treatment and for participants who were longtime users of CAM. As Allan explains in a retrospective account of how expectations changed over the course of treatment, finding hope is a significant part of the process, as is a sense of control or feeling of being able to solve one’s own problems.

*I think my expectations got broader and deeper as I started to sort of advance, I think I started to feel positive that there was some hope and I could start thinking that there was kind of light at the end of the tunnel, at least partway through and I think that helped, believing that something positive was gonna happen, instead of going in like I did at first, where I just wasn’t sure, and I was just hoping for anything. I think as I got to understand it better and patience and expectations and hopes at least got bigger, that eventually, I could solve, I guess solve more of the problems and feel better each time, each year, hoping there was a goal out there that I could attain. (Allan, Yoga participant, Tucson, Cross-sectional)*

### Realism versus hope?: Multiple evaluations of accepting chronic pain and ongoing maintenance

Over the course of the interviews, participants seemed more aware of the chronic nature of pain and the need to shift from searching for cure to seeking maintenance strategies. While awareness and control appear in the above quotations as positive, these factors imply some degree of personal responsibility for their health [45]. Willingness to accept responsibility for their own health appeared to be a factor in how participants assessed and explained changes in pain level and experience. For some participants, increased awareness and acceptance of the need for maintenance strategies and self-care was described as a successful “outcome” of treatment. For others, similar experiences were relayed with a sense of disappointment at having learned that cure was unlikely. This highlights the heterogeneity in how participants respond to expectation management and co-construction of self-care and personal responsibility or control in interactions with CAM therapists [25,38].

Cindy described her realization that managing chronic pain is a “lifelong burden”. By comparing her goal-setting to that of a marathon runner, she finds some reassurance in being able to envision the stages toward “healing”.

*[Chiropractic has] helped me … I’ve realized I’m going to have to do this for the rest of my life, the stretching, the strengthening, you know? It has made me more conscious of how I sit and how I walk. I notice that now. I notice the steps I take. …I’ve become a lot more aware of my back and pain and realize my lifelong burden is to take care of it… it’s helped me to realize and think of goals too… And it helps prepare me if I think about it mentally first, kind of like if you’re a marathon runner or whatever you know? … I’ll think of the stages that I will go through to be where I want to be on the healing. (Cindy, Chiropractic participant, Seattle, Interview 3)*

For other participants, however, increased awareness of the ongoing need for pain management, self-care, and long-term coping strategies led to decreases in hopefulness. Despite experiencing some benefit from seeing a massage therapist, Linda reported disappointment at realizing that massage would not cure her back pain. She points out, however, the realization that the pain is something she needs to attend to and which includes paying attention to posture and overall management of the pain in daily life.

*I think the one thing that you had asked me about expectations is I just really thought it would be pretty much obtained by six weeks and I don’t think it was to a level so that was a little surprising if not disappointing. But I think the only thing, I don’t know if surprise is the word, but is that I learned is that I can’t really -- and that when you have this sort of thing, you have to -- it’s a huge part of life that you have to attend to. It’s not something you can just have someone fix and then go home, you know. It’s something that it’s part of, you know, like when I sit at my desk, I have to get up from my desk. I have to kind of like move. It’s not crippling but it just needs to be managed. (Linda, Massage participant, Seattle, Interview 3)*

Helen, a long-time user of CAM, explicitly equates being realistic with feeling more negative about pain as a lifelong issue. Accepting pain as a lifelong issue requiring continuous use of maintenance strategies, in this statement, is equated with being “doomed” to it.

*I feel like the more I see [any kind of] specialist, the more I feel kind of doomed, like that I just have a screwed up back, because a lot of them will say things like, “Oh,” like they’ll think I’ll have scoliosis, or like weird physiological issues and you know, it’s made me feel a little bit like, OK, my whole life is going to be dealing with this, whereas I think early on as a runner and initially injured myself, like, 10 years ago, I didn’t think of myself as somebody who had physical issues. So now I actually feel a little more negative about, or just realistic about the fact that I’ll be dealing with this thing my whole life. (Helen, Chiropractic participant, Tucson, Cross-sectional)*

### Case example: Leslie, Yoga participant, Seattle

Although not intended to be representative of the experience of all participants in this study, Leslie provides an example of how expectations change over time in the context of ongoing attempts to establish or maintain meaning in life with chronic pain. While some of her expectations are specific to yoga, the process of adapting and revising her initial expectations in response to change over time was common among participants, regardless of therapy. Over the course of her experience with yoga, Leslie adjusts her expectations, her sense of living in her body, and her explanations about what a reduction in pain means for her overall process of moving toward “healing”. In her initial interview, Leslie expressed low expectations, explaining that she was “not an optimist” due to her experience with chronic pain. Leslie expected yoga to allow her to be proactive in managing her pain.

*I don’t [expect my life to change], I’m not an optimist by nature, certainly lost my optimism through this whole thing, I really don’t expect much. (Leslie, Interview 1)*

*I think yoga is probably one of those things that, again, for somebody who’s my age and my history, it’s proactive, it’s a proactive treatment that lets you do something about your pain… [yoga will] give me a chance to do something about it myself. (Leslie, Interview 1)*

After a few treatments, Leslie continued to express expectations about using yoga as “another tool”, admitting she didn’t “think there’s any magic bullet to it all”. She perceived a need for continued commitment to management and feared that this commitment could be undermined by “feeling a little bit better” as a result of yoga. In the second interview, Leslie reported further realization that pain reduction would not be sufficient in itself. She reiterated that a reduction in pain threatened to remove motivation for moving toward her goal of “healing”. She hypothesized, however, that *if* yoga were to reduce pain it might substitute for the pain as a motivator toward continued attention to maintenance strategies and to her body.

*I think my expectation would simply be that that yoga is another tool and I’m at a point I have enough momentum moving forward that yoga will help me build up that momentum and keep me progressing, ‘cause I think this chronic pain and chronic injury, if you go, “Oh, I feel a little bit better”. And then you sort of stay in your pattern, that’s, you don’t progress beyond, you have to constantly, constantly be pushing yourself … pain reduction doesn’t actually mean that you’ve fixed your problem, until you’re able to do the things you should be … so a reduction in pain isn’t my goal, my goal is healing, and so my expectation of yoga is simply that it would be something to keep me moving forward at each level of progress. (Leslie, Interview 2)*

In a final interview, after 6 yoga classes, Leslie reported having learned that pain is not a constant state, but rather can “ebb and flow”. This realization appeared to impart a renewed sense of hopefulness with regard to the future.

*I think I’ve learned that even though I’ve had this pain for so long that it feels really permanent and impermeable, I think I’ve learned that it can ebb and flow and it can be less painful and it can be like half the days in bad days instead of only ever having bad days. So that’s been like probably a good thing to learn. Just that the pain is malleable, but it’s changeable. I haven’t felt like that in a very long time. (Leslie, Interview 3)*

## Discussion

Interview narratives are not simply accounts of inner thoughts or feelings, but rather verbal processes in which individuals attempt to make sense of experiences and of their reactions [46,47]. At a time when participants were actively seeking a novel treatment for pain, CAM care may have provided the additional benefit of assisting with attaining or regaining a sense of control, increased awareness, and a stance of acceptance toward the long-term implications of living with chronic low back pain.

Although some participants were already aware of the importance of maintaining their own health, even those participants who expressed acceptance of the need for strategies to maintain overall health and for manage chronic pain at baseline reported greater awareness of the need for these strategies and increased empowerment to make life changes at the three month follow-up interview. Sasagawa et al. [23] raised questions about whether participants seeking CAM were already more motivated and engaged in their own care than patients who did not seek CAM, or whether CAM increased their motivation. Our findings suggest the latter explanation may be true. Even those participants who were disappointed that the treatment did not result in improvements in key outcome domains expressed greater awareness of the need for self-care and of interconnections between pain and broader lifestyle factors.

We observed that among the participants we interviewed, evaluations of treatment outcomes were contingent not only on initial expectations, but also on whether or not participants viewed their low back pain as “chronic” or as something that could potentially be resolved or cured. There is a significant body of research on the potential therapeutic value of accepting the presence of chronic pain [48-50]. These researchers report that acceptance of the chronic nature of pain was associated with whether patients adopted effective or ineffective coping strategies [49,51] as well as with better emotional, social and physical functioning [50]. Acceptance of pain often entails re-framing a new life in the present rather than attempting to return to a past version of “normal” [52] as well as acknowledgment that cure is unlikely and a shifting focus from pain to non-pain aspects of life [51,53]. Huet and colleagues [54] reported that individuals only came to a point of acceptance after a process of grieving and regret. As Jackson [55] points out, individuals with chronic pain often view their pain as indicative of bodily damage. Experiencing pain that lasts longer than expected often leads individuals to seek diagnosis, legitimacy, and meaning, which can come to dominate individuals’ lives [56] until they are eventually confronted with a discourse of adaptation that implores them to consider acceptance of life with pain [51]. In our analysis of interviews with patients about their expectations for treatment, we observed that narratives initially focused on whether or not the treatment could lessen pain or improve ability to participate in meaningful activities impacted by pain. In later interviews, emphasis shifted to be broader in focus, a shift from focus on pain to non-pain aspects of life that align with the features of accepting pain as described by Risdon et al. [51].

Thorne et al. [26] suggest that CAM therapies themselves are part of a complex process of self-care management. Interviews with CAM practitioners suggest that the practitioners see themselves as actively promoting self-responsibility and attention to the need for self-care [38]. Our findings with patients further corroborate this idea and are consistent with suggestions that interactions with CAM practitioners may contribute to positive behavior change, including an increased acceptance of personal responsibility for managing pain [30]. Thus, our findings contribute to a growing body of evidence suggesting that CAM may have the potential to increase patients’ awareness of connections between lifestyle, mind, and body and to lead to perceptions of increased control over pain and self-efficacy for managing pain [7,26]. Baarts and Pedersen [57] found that no matter whether CAM treatment was “successful”, their interviewees reported increases in general well-being. They observed that this led to increased motivation to care for the self and body and not to dependency on a CAM practitioner.

These interviews were conducted as part of a study ultimately aimed at creating a questionnaire to measure participants’ expectations in CAM clinical trials. Interviews focused on prior experience, beliefs about illness causation, attitudes toward the possibility of finding relief, and on expectations and hope for the treatment’s outcome. Unfortunately, we did not ask participants about their expectations about the likelihood of self-care recommendations prior to treatment. Therefore, detailed analysis of their expectations about the likelihood of self-care recommendations prior to treatment and of others aspects of participants’ experience with treatment (e.g., their satisfaction with more conventional treatments, changing beliefs about healing in general) is beyond the scope of the data. In addition, longitudinal interviews were conducted over a period of three months and use of CAM during that time period varied substantially, but tended to be less than what clinical trials offer. Interviews with long-term CAM users also included participants whose CAM use for back pain varied over the course of their lives. Participants were seeking multiple CAM therapies from different practitioners on their own. We are unclear how our findings would compare to persons seeking CAM for other conditions, which may differ in their natural history. Some researchers have reported, however, that the level of self-care and emphasis on empowerment and self-responsibility we observed is unique to CAM [25]. Because of the relatively small sample size and community based recruitment strategy, we focus on findings that were observed across all four therapies among back pain patients and therefore we do not comment on differences among therapies. Future research should consider not only how expectations change over time, but also how feelings of empowerment and control may require accepting a conception of wellbeing not centered around the possibility of cure.

## Conclusions

Participants seeking one of four CAM therapies focused most of their expectations and hopes during initial interviews on whether or not the treatment could lessen their pain. In later interviews, where self-care was included in the interview guide, emphasis tended to be broader, shifting from needing to be “fixed” to an emphasis on the body as a work in progress, in need of ongoing attention and care from both the patient and the CAM practitioner. This trend toward attention to management, self-care and wellness, and increased acceptance of pain as chronic, was reported by participants seeking all four therapies. We also noted that this overall shift over time was evaluated in various ways by participants, depending on how they were able to orient their sense of self and understanding of illness toward ongoing management and engagement.

These findings suggest the value of further research into the potential of the CAM therapeutic process to assist patients in taking control of their health management and wellness. Sointu [58] argues that CAM use is becoming increasingly common because the “discourse of well-being” (9) embedded in CAM interactions (see also [24,25]) resonates with how people understand their bodies and selves. Our data support the finding that CAM participants report greater awareness of the need for ongoing engagement in their own care, an increased sense of control or empowerment, and motivation to seek effective coping strategies. A reconceptualization of healing to include recognition of the interconnectedness between health and lifestyle factors and of the lifelong need to seek well-being was reported among participants, regardless of whether treatment was perceived as “successful”. Further research should consider how these attitudes and beliefs change over time, whether they are associated with important changes in behavior, and if so, what role CAM practitioners may play in supporting a positive view of changing ideas about self-care and healing.

## Abbreviation

CAM: Complementary and alternative medicine
